# Apple-Harvesting Robot Based on the YOLOv5-RACF Model

**DOI:** 10.3390/biomimetics9080495

**Published:** 2024-08-14

**Authors:** Fengwu Zhu, Weijian Zhang, Suyu Wang, Bo Jiang, Xin Feng, Qinglai Zhao

**Affiliations:** 1 College of Engineering and Technology, Jilin Agricultural University, Changchun 130118, China; zhufengwu@jlau.edu.cn (F.Z.); zwj05252021@163.com (W.Z.); wang_suyu1218@163.com (S.W.); 2 Kunyu Intelligent Control (Jilin) Technology Co., Ltd., Changchun 130118, China; jiang18943986899@163.com (B.J.); feng18501383999@163.com (X.F.)

**Keywords:** YOLOv5, apple harvesting, autonomous navigation, object detection, image processing

## Abstract

To address the issue of automated apple harvesting in orchards, we propose a YOLOv5-RACF algorithm for identifying apples and calculating apple diameters. This algorithm employs the robot operating dystem (ROS) to control the robot’s locomotion system, Lidar mapping, and navigation, as well as the robotic arm’s posture and grasping operations, achieving automated apple harvesting and placement. The tests were conducted in an actual orchard environment. The algorithm model achieved an average apple detection accuracy (mAP@0.5) of 98.748% and a (mAP@0.5:0.95) of 90.02%. The time to calculate the diameter of one apple was 0.13 s, with a measurement accuracy within an error range of 1–3 mm. The robot takes an average of 9 s to pick an apple and return to the initial pose. These results demonstrate the system’s efficiency and reliability in real agricultural environments.

## 1. Introduction

High-value crops, such as apples, oranges, and bananas, are widely cultivated globally and are a crucial part of the daily diet, playing a key role in international trade. However, with advancements in agricultural practices, traditional manual harvesting methods can no longer meet the increasing demand. Additionally, the aging labor force in rural areas, combined with the migration of younger people to urban areas, has led to a labor shortage and rising production costs [1,2]. In this context, the research and development of harvesting robots have become an urgent need to address labor shortages and reduce production costs. The emergence of harvesting robots not only ensures the timely harvesting of fruits in the absence of labor but also improves harvesting efficiency and product quality. This not only increases the yield and quality of orchards but also reduces labor costs and intensity, bringing practical economic benefits to farmers. Therefore, harvesting robots play an important role in solving labor shortages and reducing production costs in agricultural production. As early as the 1960s, Sisler [3] began attempting to design fruit-harvesting robots. With technological advances, particularly in electronics, information technology, and computer science, especially artificial intelligence, fruit-harvesting robots have become increasingly intelligent and modernized. In recent years, the development of robotic arms for fruit-harvesting robots has mainly focused on the design of end effectors, path planning, and the development of vision systems. In the visual recognition part of harvesting robots, more and more experts and scholars are choosing the You Only Look Once (YOLO) model for recognition and detection instead of traditional algorithms. This is because YOLO has the advantages of fast recognition speed, high recognition accuracy, less susceptibility to lighting conditions, and strong robustness [4,5,6]. It has been successfully applied to many harvesting robots. Gao et al. [7] combined YOLOv4 with a particle filter algorithm and a robotic arm for grasping moving objects, achieving a success rate of up to 88%. Miao et al. [8] also used the deep learning method for the identification and positioning of tomatoes, then proceeded to grasp them. The results showed an average grasping time of 9 s with an error of only 2 mm. Yu et al. [9] proposed a novel strawberry-harvesting robot equipped with a fruit posture estimator named rotational YOLO (R-YOLO). By providing additional rotation angle annotations for the dataset, the accuracy of fruit positioning was improved. However, its limitation lies in the high time and labor costs of manually adding extra annotations. Many fruit-harvesting robots developed based on the YOLO algorithm have been applied to the harvesting of lychees, tomatoes, strawberries, and other fruits [10,11,12]. These harvesting robots have verified the effectiveness of the YOLO algorithm in fruit recognition. Therefore, this paper chooses YOLOv5 for target localization and recognition in its vision system. Hiroshi and Wei Guo [13,14] used thermal imaging cameras, hyperspectral cameras, and ultraviolet cameras to capture images of fruits, reducing the impact of natural light. However, the high equipment costs make it difficult to apply them in agricultural harvesting robots. Kanae et al. [15] developed a cherry-harvesting robot with a camera mounted on a robotic arm, allowing it to rotate 360 degrees to avoid blind spots. After capturing the images, the camera sends them to a computer for analysis, and the results are transmitted to the upper-level controller to control the robotic arm for harvesting. Hayashi et al. [16,17] developed a mobile harvesting robot using a visual sensing system to capture images. It uses three cameras, two mounted on the sides of the robotic arm to determine the target’s positional information, and one in the middle to determine the tilt angle. Due to the complex operating environment and uneven natural light, successful harvesting was not achieved. In the context of improving human work efficiency, Williams et al. [18] identified kiwifruit using machine vision and used a four-fingered robotic arm for grasping, enhancing the efficiency of the grasping process. Berenstein and Edan [19] proposed a human robot collaboration framework that combines robotic spraying tasks with manual target detection, improving the accuracy of vineyard spraying and reducing material usage by 50%. To address the issue of uncertain path trajectories generated by most high-degree-of-freedom robotic arms during movement and grasping, Song et al. [20] proposed a trajectory planning method based on RBF neural networks. By combining neural networks with robotic arm trajectory path planning, simulation results demonstrated improved response and tracking performance of the robotic arm, ensuring smooth operation during the trajectory planning process. Similarly, Liu et al. [21] employed an adaptive bias radial basis function neural network (RBFNN) control method, ensuring that the robotic arm maintains high control accuracy under different load conditions. Another important aspect of harvesting robots is the design of the end effector. Due to the complex environment of actual agricultural scenarios, where the harvesting targets may be occluded, it is necessary to design different end postures to improve the success rate of target grasping. Different types of end effectors have been designed according to different harvesting targets, such as common gripper-type [22] and vacuum suction-type [23] end effectors, to ensure efficient fruit harvesting while avoiding damage to the target. Yaguchi et al. [24] developed a tomato-harvesting robot using a two-finger surface gripper with infinite rotational joints, achieving an average harvesting speed of 23 s per fruit. However, when tomato clusters are very dense, the gripper may grasp multiple fruits, and the calyx may be damaged if the stem axis is deep within the rotational axis. Knodo et al. [25] developed a tomato-harvesting robot where the robotic arm first uses an end effector to cut the fruit from the tree and then uses pneumatic principles to suck the fruit into a tube. Xiong et al. [26] designed a strawberry-harvesting robot with a high fault tolerance and a non-contact mechanism. The robot’s gripper integrates sensors that can detect and adjust any positional misalignment. It also features an internal compartment for collecting strawberries, significantly reducing the time required for harvesting. Liu et al. [27] discussed different tomato harvesting modes (stretching, bending, rotating) and various theories of force transmission, deformation, and fatigue fracture during the harvesting process in detail, aiming to reduce harvesting damage and improve harvesting efficiency.

In the field of apple-harvesting robots, many scholars have conducted extensive work. Stajnko et al. [28] utilized thermal imaging to capture apple images to reduce the impact of natural light. They then employed image processing techniques to extract apple image features for quantity and diameter estimation, achieving high accuracy. However, thermal imaging datasets are relatively difficult to obtain. Tao Li et al. [29] used a deep learning segmentation network for apple recognition and localization, as well as size estimation in cases of partial occlusion. Experiments showed that the deep learning method could reduce the positioning error by an average of 43%. Wei Chen et al. [30] improved YOLOv4 to enhance apple detection performance in complex environments, proving that the method performed well under conditions of occlusion and lighting changes, outperforming traditional methods. These methods for apple visual recognition and localization lay the foundation for apple harvesting. Zhao Zhang and Paul H. Heinemann [31] implemented a low-cost apple-harvesting robot that reduced the time and labor costs required by traditional harvesting methods, thereby improving harvesting efficiency. However, the design process of the vision system was cumbersome. The apple-harvesting robot introduced in Zhang’s paper [32] also utilized fluid pipelines and pneumatic principles to pick apples. In contrast, Wang et al. [33] developed a more novel and advanced apple-harvesting robot gripper, consisting of four conical soft fingers and a suction cup. After the fingers grasp the apple, the suction cup attaches to the target, and the claws close, rotating and pulling to pick the apple from the tree. This gripper increased the success rate of harvesting and reduced apple damage.

This paper proposes an apple-harvesting robot that integrates multiple technologies. The robot uses various sensors, including radar, inertial sensors (IMU), and vision sensors. By fusing data from multiple sensors, the robot’s automation level is enhanced, ultimately achieving autonomous harvesting and harvesting without human intervention. The general contributions include the following:

1. We propose a YOLOv5-RACF algorithm that combines YOLOv5 for target detection, utilizes the random sample consensus (RANSAC) algorithm for apple contour fitting, and employs a custom activation function to filter outliers to determine the diameter of apples. The objective is to control the opening and closing angle of the robotic arm gripper, reduce mechanical damage during fruit harvesting, and achieve fruit size classification.
2. We intend to equip the robot’s autonomous navigation hardware with Lidar and an inertial measurement unit (IMU) inertial sensor and utilize multiple algorithms to achieve high-precision map construction and navigation obstacle avoidance. These algorithms include the gmapping algorithm for creating high-precision maps of closed environments, and the Dijkstra, A*, and timed elastic band (TEB) algorithms for navigation and obstacle avoidance.
3. We aim to efficiently integrate the robot’s grasping system with the autonomous navigation and obstacle avoidance system within the ROS framework. Through ROS communication mechanisms, the system achieves simultaneous apple picking while autonomously navigating and avoiding obstacles. This system not only enhances the robot’s environmental perception and path planning capabilities but also improves its efficiency in autonomous operations in complex environments. To validate the effectiveness of the picking robot system, orchard trials were conducted.

The remainder of the paper is organized as follows. Section 2 introduces the YOLOv5 model for apple recognition and localization, including data acquisition and training. It details how the combination of a depth camera and the YOLOv5 algorithm achieves accurate apple target identification and localization, and the use of the RANSAC algorithm with a custom nonlinear function for apple diameter measurement. This section also covers the gmapping, Dijkstra, A*, and TEB algorithms for autonomous navigation, as well as integration and communication methods within the ROS framework. Section 3 presents experimental analysis and validation, showing that the YOLOv5n model and the integrated apple-harvesting robot system under ROS demonstrate high accuracy and efficiency. Section 4 summarizes the research findings and explores future research directions, concluding that the combination of the YOLOv5-RACF model with a depth camera significantly improves the accuracy of apple recognition, localization, and diameter measurement.

## 2. Materials and Methods

### 2.1. Experimental Hardware and Software System

**Figure 1:**
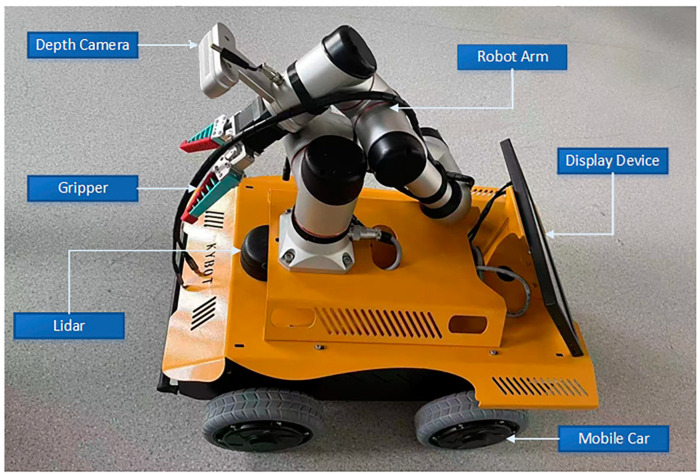
Schematic diagram of the apple-harvesting robot.

In the model training section, we operate using the Windows 10 64-bit operating system, utilizing a 12th Gen Intel(R) Core(TM) i7–12700F 2.11 GHz CPU and an NVIDIA GeForce RTX 4070 GPU with 12 GB VRAM. The research is based on the PyTorch deep learning framework. The development environment includes PyTorch 1.11.0, CUDA 11.3.1, and Python version 3.8.16, used for training and validating the YOLO algorithm. The robotics part includes a four-wheeled mobile chassis (equipped with a power supply and inertial sensors (IMU)) for robot movement and the installation of a robotic arm; the chassis is produced by Kunyu Intelligent Control Technology Company Limited, Changchun 130118, China. A Jetson AGX Orin development board with 32 GB memory was used for deploying robot algorithms and conducting robot experiments; the Jetson AGX Orin is a high-performance, low-power embedded AI computing platform launched by NVIDIA. It features an NVIDIA Ampere architecture GPU, delivering up to 200 TOPS (trillion operations per second) of AI inference performance, capable of handling complex neural networks and AI algorithms. Equipped with 8 high-efficiency Arm Cortex-A78AE CPU cores, it offers excellent computing power and multitasking capabilities. It provides various I/O interfaces, including USB 3.2, PCIe, GPIO, I2C, and SPI, facilitating connections to various external devices and sensors. With adjustable power modes ranging from 15 W to 60 W, it achieves optimal performance and power balance for different application scenarios. The powerful computing capability of the AGX Orin development board is sufficient to support the deployment of YOLOv5 and navigation tasks. An RPLidar A2 laser radar for robot obstacle avoidance and autonomous navigation, namely RPLidar A2, with its high precision, full 360-degree scanning, high sampling rate, and low power consumption, has been widely used in several fields, such as robot navigation, map construction, and environmental perception. It is capable of supporting orchard environment scanning and mapping. An Elite six-degree-of-freedom robotic arm equipped with a flexible gripper was used, as shown in Figure 1. The Elite robotic arm uses high-precision servo motors and advanced control algorithms to ensure motion accuracy and repeatable positioning, making it suitable for applications that require high-precision operations. Its design allows for multi-angle and multi-directional operations, adapting to various complex working environments. It supports multiple control interfaces and communication protocols, facilitating integration with other devices and systems to achieve coordinated work among multiple devices. These features make it well-suited for the task of apple picking in orchards. The robot software system uses Ubuntu 20.04 equipped with a ROS environment that supports both Python and C++. Additionally, it includes drivers and ROS packages for a depth camera, Lidar, and IMU to enable various robot functionalities.

### 2.2. Data Acquisition and Enhancement

**Figure 2:**
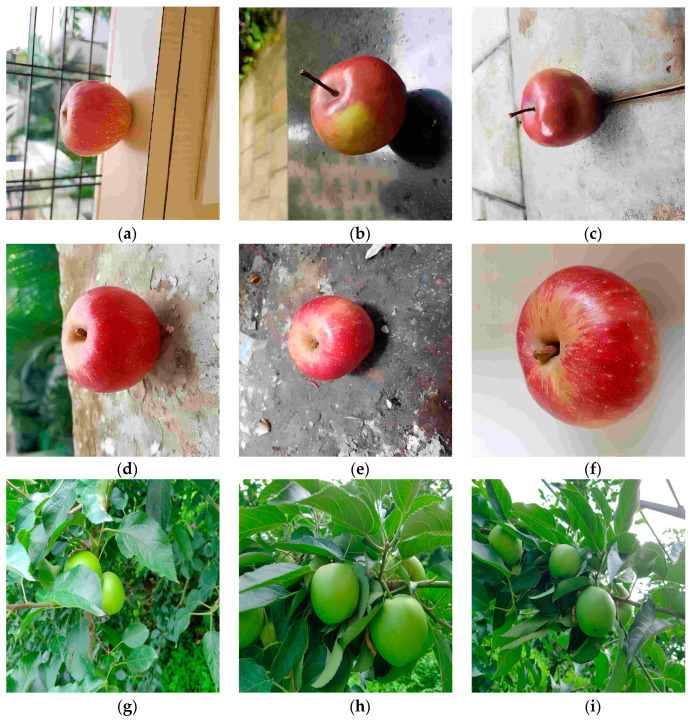
Images of apples. (**a**) An apple on a windowsill; (**b**) An apple on a rough surface; (**c**) An apple resting on a smooth surface; (**d**) An apple placed on a stone surface; (**e**) An apple on a dirt ground; (**f**) A close-up of a single apple; (**g**) A cluster of green apples on a tree; (**h**) Green apples growing among leaves; (**i**) A bunch of green apples on a branch.

The dataset for this study is derived from two sources: online resources and natural orchard conditions. From the online resources, we selected a total of 2044 apple images under various lighting conditions, with clear quality, and with both indoor and outdoor backgrounds at different angles. These images were stored in the JPG format. To enrich the dataset and meet the requirements of real-time detection by robots in complex agricultural scenarios, we collected additional data in July 2024 at the orchard base of Jilin Agricultural University in Changchun, Jilin Province, China. The collection device was an iPhone 13 smartphone, generating images with a resolution of 4096 × 3072 pixels, also stored in the JPG format. To ensure the randomness and diversity of the images, 1026 orchard apple images were obtained from different angles, different sizes, and various levels of occlusion and backgrounds in a complex orchard environment, as shown in Figure 2.

**Figure 3:**
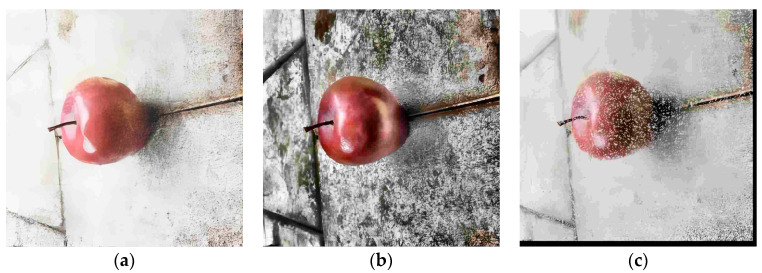

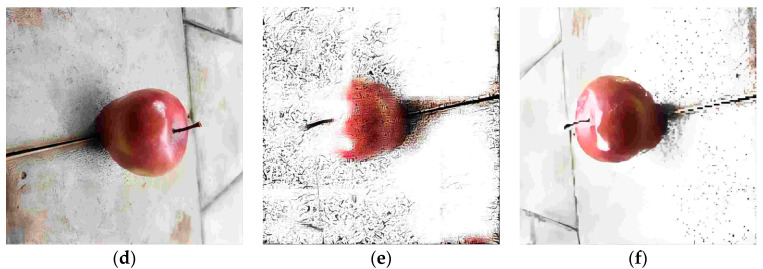
Augmented apple images. (**a**) Add and noise result; (**b**) CLAHE result; (**c**) Salt and transl result; (**d**) H-flip and v-flip result; (**e**) Dropout and edge result; (**f**) M-pool and edge result.

After splitting the dataset in a 9:1 ratio, we further enhanced it to increase the model’s robustness and the accuracy of recognition. Enhancements included adding Gaussian and salt-and-pepper noise to simulate the blur caused by camera shake, implementing vertical and horizontal flips to broaden the viewing perspectives, applying histogram equalization to highlight apple features, and expanding the brightness of images to improve recognition stability under different lighting conditions as shown in Figure 3. After augmentation, the dataset comprises a total of 9934 apple images. To reduce the occurrence of overfitting in the dataset, we chose to first divide the dataset and then perform augmentation. Additionally, each augmentation employs two different data augmentation methods simultaneously to reduce similarity to the original dataset. Additionally, we manually annotated the original images using labeling and set the label name to goodapple. The specific details of the training are presented in Table 1.

### 2.3. YOLOv5

**Figure 4:**
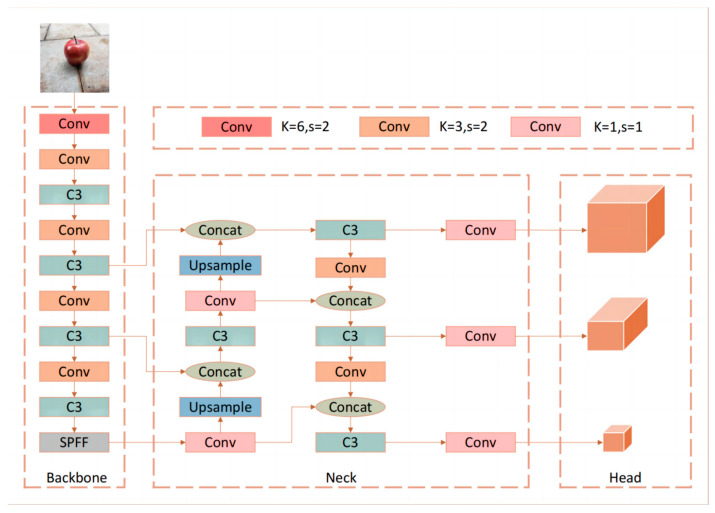
Yolov5 model.

To identify and locate apple targets, YOLOv5 was utilized. This network is the fifth iteration of the YOLO series and not only surpasses its predecessors (YOLOv4) in detection accuracy but also boasts a compact model that saves computational power, making it suitable for mobile deployment. Its architecture is divided into three main parts: the backbone network, neck network, and head network, each with their own distinct function. The backbone network is responsible for feature extraction from the input images. In addition to conventional convolutions, it incorporates the CSP (cross-stage partial) structure; the CSP structure is applied in the C3 modules in the diagram below. This divides the feature map into sections before merging them through a cross-stage structure. This method significantly reduces redundant computations, decreases the computational load, and enhances the fusion of features across channels. The neck network is responsible for the fusion of shallow features (such as the color, texture, shape, and contour of apples) with deep semantic features. It adopts the FPN (feature pyramid networks) structure and has been improved to enhance the feature fusion capability. The FPN structure is applied in the neck part of the diagram below to enhance the feature fusion capability. Next, the head network uses the fused features from the neck network to predict the bounding boxes and class probabilities for each apple detection object, essentially being responsible for the final localization and classification of objects within the image. The network model is shown in Figure 4.

### 2.4. Apple Target Localization

Firstly, the D435i depth camera’s packages and drivers are configured in the ROS system. Following this, the depth camera captures the apple target within the field of view, and the trained YOLOv5 network is used to identify the apple. The pixel coordinates of the top-left corner of the bounding box are defined as (xmin, ymin), and the pixel coordinates of the bottom-right corner are (xmax, ymax). Upon detecting the apple target, its pixel center point (px, py) is obtained. By subscribing to the camera intrinsic parameters’ topic, the camera’s intrinsic parameters are acquired. The camera intrinsic parameters include the image center coordinates (ppx, ppy) and the focal length parameters (fx, fy). The apple’s two-dimensional coordinates (x, y) in the camera coordinate system are calculated. These two-dimensional coordinates are then fused with the depth of the apple’s pixel center point obtained through subscription (z), resulting in the three-dimensional spatial coordinates of the apple in the camera’s coordinate system. The pixel center point and the three-dimensional coordinates of the apple are calculated using the following formulas:

(1) px=(xmin+xmax)/2

(2) py=(ymin+ymax)/2

(3) x=(px−ppx)/fx×z

(4) y=(py−ppy)/fy×z

After obtaining the current frame’s three-dimensional spatial coordinates of the apple, we average the coordinates over 10 consecutive frames to determine the final three-dimensional spatial coordinates of the apple. This approach smooths and reduces the impact of noise present in individual frame data, thereby improving the localization accuracy and smoothing short-term measurement fluctuations to make the results more stable. The calculation formulas are as follows:

(5) $$\bar{x}=\frac{1}{10}\sum_{i=t-9}^{t}x_{i}$$

(6) $$\bar{y}=\frac{1}{10}\sum_{i=t-9}^{t}y_{i}$$

(7) $$\bar{z}=\frac{1}{10}\sum_{i=t-9}^{t}z_{i}$$

where $\bar{x}$, $\bar{y}$, and $\bar{z}$ are the averaged coordinates, x_i_, y_i_, and z_i_ are the coordinates of the apple in the i-th frame, and t is the index of the current frame.

### 2.5. Apple Size Calculation Method

**Figure 5:**
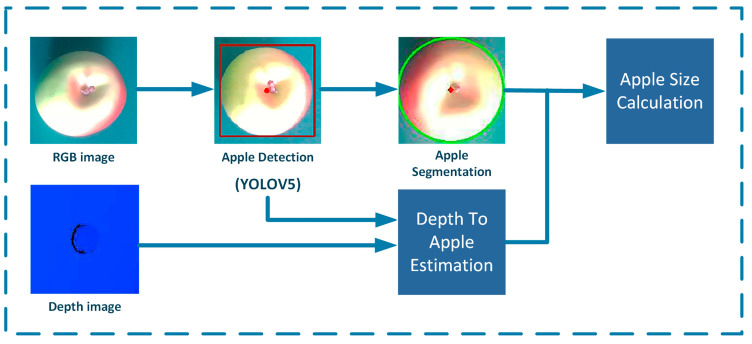
Apple size estimation process.

Accurate estimation of fruit quantity and size is crucial, as it helps farmers determine the workload required for harvesting, the number of fruit boxes, and storage space. It also enables quality classification based on different sizes. For autonomous harvesting robots, determining the diameter size of apples allows for the control of the robot’s gripper opening and closing angles in subsequent studies, thereby reducing mechanical damage during apple harvesting. Before obtaining the size of the apples, the most crucial step is to segment the apple targets. Many researchers have conducted in-depth studies in the direction of target segmentation. Ma et al. [34] used an improved kernel metric weighted fuzzy C-means algorithm for image segmentation and utilized a seed filling algorithm to find the maximum connected region, retaining the main features to reduce noise impact. Lehnert et al. [35] used the Kinect Fusion algorithm along with color segmentation and clustering techniques to segment sweet pepper objects. Rizon et al. [36] first utilized morphological operations to identify the target, and then used the randomized Hough transform to recognize the contours. Compared to the traditional Hough transform, this method improves detection accuracy and reduces computational load. Sun et al. [37] simulated the principle of sea level submerging islands to segment white button mushrooms and their background soil and combined it with the Hough transform to measure the cap diameter. The results showed that the diameter measurement error of this method was 4.94%, with an average processing time of 0.5 s per mushroom. Firstly, this paper detects apple targets using YOLOv5, and then the bounding box containing the apple target is extracted for image processing. Compared to directly using traditional methods to extract the apple contour, recognizing the apple target with YOLOv5 first and then extracting the bounding box containing the apple image can significantly reduce the influence of irrelevant background and accelerate the computation. The preprocessing of apple images begins with grayscale conversion, transforming the three-channel RGB apple image into a single-channel grayscale image. This focuses more on the apple target area and applies the CLAHE algorithm to enhance image contrast. Subsequently, Gaussian blur is used on the grayscale image to reduce apple image noise and details, making contour extraction more reliable by smoothing the image. After obtaining the edge information, the Canny edge detection method is used to identify the edges of the apple image, as shown in Figure 5. With the contour points coordinates obtained, the RANSAC algorithm and a custom function are used for circle fitting of the apple contour and outlier filtering. Compared to the traditional least squares fitting method, the proposed method in this paper is more robust to outliers and noise. The calculation formulas are shown as follows.

The equation of the circle is represented as follows:

(8) $$(x-a{)}^{2}+{\left(y-b\right)}^{2}=r^{2}$$

where (a, b) is the center of the circle, and r is the radius.

To determine a circle, at least three points are needed. Let these points be (x_1_, y_1_), (x_2_, y_2_), and (x_3_, y_3_). The solution can be obtained through the following system of linear equations:

(9) AX=B

(10) $$A=\left[\begin{array}{ccc} x_{1} & y_{1} & 1\\ x_{2} & y_{2} & 1\\ x_{3} & y_{3} & 1 \end{array}\right],\;B=\left[\begin{array}{c} x_{1}^{2}+y_{1}^{2}\\ x_{2}^{2}+y_{2}^{2}\\ x_{3}^{2}+y_{3}^{2} \end{array}\right]$$

Solving the above system of linear equations, the solution X is as follows:

(11) $$X=\left[\begin{array}{c} D_{x}\\ D_{y}\\ C \end{array}\right]$$

Thus, the center (a, b) and the radius r can be expressed as follows:

(12) $$a=\frac{D_{x}}{2},\;b=\frac{D_{y}}{2},\;r=\sqrt{C+a^{2}+b^{2}}$$

For each point (x_i_, y_i_), we calculate its distance to the fitted circle center (a, b) as follows:

(13) $$d_{i}=\sqrt{(x_{i}-a{)}^{2}+(y_{i}-b{)}^{2}}$$

If ∣d_i_ − r∣ < ϵ, the point is preliminarily considered a valid fitting point. We repeat the process 1000 times, and then use a custom nonlinear function for precise fitting. This function assigns different weights to fitting points and outliers, retaining points with activation values greater than 0.5. The formula is as follows:

(14) $$Y=\frac{1}{1+e^{\alpha (d_{i}-r)}}$$

where d_i_ is the distance from each point to the circle center, r represents the radius of the fitted circle, α controls the steepness of the function.

### 2.6. Laser-Based 2D Navigation Algorithm

To meet the experimental requirements, a Lidar system was used for mapping and autonomous navigation. After comparison and selection, the gmapping algorithm was chosen. This algorithm uses particle filter technology to separate map creation and localization. Its advantages include low computational demand and relatively high accuracy. However, its limitations are that it is only suitable for small to medium-scale environments. During the autonomous navigation process, the robot first selects a global path algorithm, Dijkstra, to generate an optimal path from the robot’s current position to the target location. The Dijkstra algorithm is a classic global path algorithm, but it is not suitable for larger-scale path planning, as it has high spatial complexity and a poor adjustment ability in dynamic environments. To overcome these shortcomings, the A* algorithm and the TEB (timed elastic band) local path algorithm were combined. The former introduces a heuristic function, making the global path search more efficient, while the latter ensures that the robot can dynamically avoid obstacles, such as suddenly appearing people or objects. Other improvements include the combination of multiple cost map layers, such as the static map layer, obstacle layer, and inflation layer, providing more accurate environmental information and more precise path planning. The main parameters of the path-planning algorithm are shown in Table 2 below. The global cost map is represented as “/map”, indicating that the global map coordinate system is a static reference coordinate system. The global map is static, representing the entire environment map used for global path planning. The local cost map is set as “/odom”, representing the odometry coordinate system, which is dynamic and changes with the movement of the robot. Due to issues, like wheel slippage, in wheeled robots, there is some error in the odometry data, which leads to errors in the robot’s position and orientation. Therefore, an IMU sensor is used to provide calibrated odometry data to help compensate and correct these errors, improving localization accuracy. As shown in Table 2, some key parameters include dt_ref and dt_hysteresis, which determine the reference value of the time step and the hysteresis time to ensure the smoothness and real-time performance of path planning. Max_vel_x and max_vel_theta define the robot’s maximum linear and angular velocities, ensuring stability during movement. Acc_lim_x and acc_lim_theta set the acceleration limits for the robot to prevent instability caused by sudden changes in speed. Yaw_goal_tolerance and xy_goal_tolerance define the allowable error range when the robot reaches the target point, ensuring precise localization. Inflation_dist and inflation_radius are used for setting the distance and radius of obstacle inflation, ensuring safe obstacle avoidance for the robot. Weight_kinematics_forward_drive and weight_kinematics_turning_radius are used to optimize the robot’s kinematic model, ensuring efficiency in forward movement and turning during path planning. Update_frequency and publish_frequency set the update frequency for global and local cost maps and the publishing frequency of ROS topics to ensure data timeliness and system responsiveness. Cost_scaling_factor and weight_obstacle set the scaling factor for the cost map and the weight of obstacles to optimize obstacle avoidance behavior in path planning. These initial values are based on the default values provided by extensively tested ROS packages and were subsequently adjusted through actual experiments to suit specific situations.

**Figure 6:**
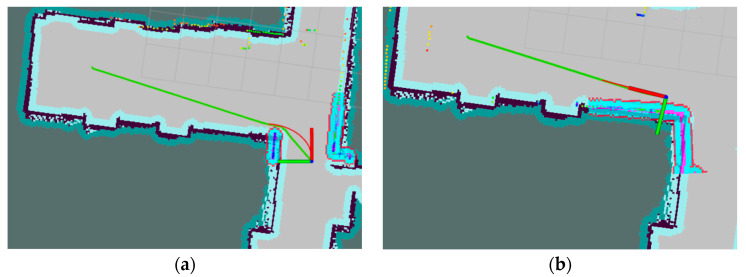
Robot navigation path diagram. (**a**) The robot’s initial path planning with LiDAR-based obstacle avoidance and local path adjustment at the corner; (**b**) The robot follows the predetermined route to the destination.

The mapping and autonomous navigation using Lidar are shown in Figure 6. The robot’s initial position is represented by the coordinate frame. The planned global route is depicted with a green line, while the local path is indicated with a red line. The robot navigates along these paths to reach the specified target location.

### 2.7. Coordinate System Transformation Based on the ROS System

**Figure 7:**
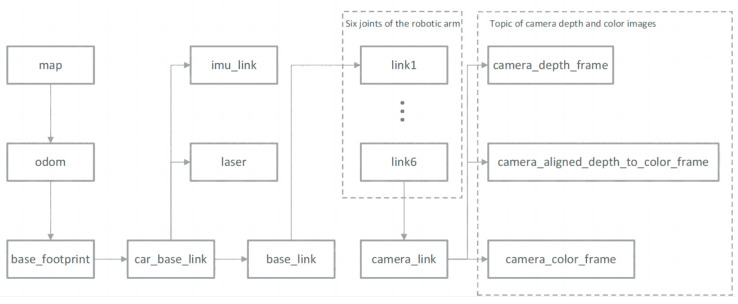
The tf tree.

In ROS, tf is used for coordinate transformations between different parts of a robot, abstracting each component of the robot into individual three-dimensional coordinate frames. It primarily consists of a broadcaster and a listener, which are responsible for broadcasting translational and rotational transformations between coordinate frames, thereby determining the transformation relationships and relative positions of various components. Each node frame represents a coordinate frame, and arrows indicate transformations between frames. The ‘map’ acts as the global coordinate frame, serving as the parent node for other coordinate frames; ‘odom’ represents the robot’s odometric frame, used to track the robot’s position and movement in space; ‘base_footprint’ represents the robot’s physical base coordinate frame, which links various sensor coordinate frames, such as the inertial sensor frame (imu_link), radar coordinate system (laser), the coordinates of each joint of a six-degree-of-freedom robotic arm (link1-link6), the depth camera coordinate frame (camera_link), etc., as shown in Figure 7. Through tf transformations, the transformation relationship between the camera coordinate system and the robot’s base coordinate system is obtained, and the coordinates of the apple in the base coordinate system are broadcasted and listened to.

### 2.8. Integration of the Navigation System for the Harvesting Robot

**Figure 8:**
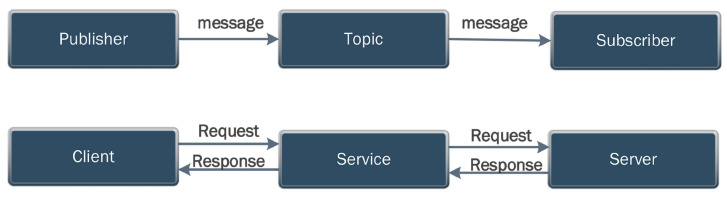
Two communication methods in ROS.

In ROS, there are two main communication methods for interaction, as shown in Figure 8. The message communication model uses a publisher to publish message content and publishes the message to a topic at a certain frequency. The topic acts as a communication channel between the publisher and subscribers, containing the message content. Subscribers subscribe to the topic, receiving the messages published by the publisher. This method enables asynchronous communication between nodes, allowing publishers to send messages at any time and subscribers to receive messages simultaneously at any time. In this paper, it is mainly used for sensor data collection and status updates, which do not require immediate responses. However, due to the nature of asynchronous communication, it cannot guarantee that subscribers receive every message. Another communication method is through a client sending a service request. The service receives the request and forwards it to the server. The server processes the request and returns a response, informing the client of the request’s outcome. This is a synchronous communication method, where the client waits for the server’s response after sending the request to confirm whether the service request has been successfully processed. In this paper, this synchronous communication method is used for coordinating the functions of various modules in the harvesting robot’s overall testing experiments. This mechanism ensures that each module operates in an orderly manner, enabling efficient autonomous navigation, automatic identification and localization, and autonomous harvesting. For example, after the client requests navigation to a specific location, the server processes the path planning and returns the navigation result. When the client requests the image processing module to locate and identify apples, the server processes the request and returns the result. Similarly, when the client requests the robotic arm to perform the harvesting operation, the server plans the arm’s joint movements to achieve the harvesting goal and returns the result indicating whether the planning was successful.

**Figure 9:**
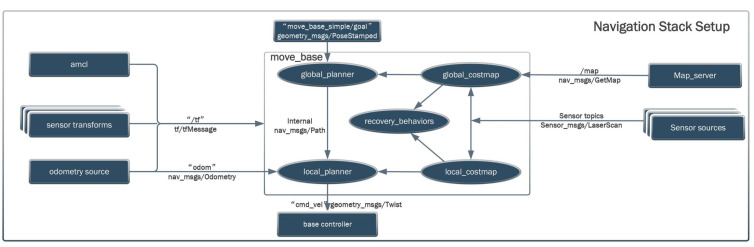
Integration of the harvesting robot navigation system.

As shown in Figure 9, this diagram illustrates the relationships and data flow between various components of the harvesting robot’s motion system. The “amcl” component uses adaptive Monte Carlo localization to determine the robot’s position on the map, estimating its current pose using sensor data and maps constructed by gmapping. “Sensor transforms” handle the coordinate transformations between different sensors, using tf to broadcast and listen to transformation information. The “odometry source” provides odometry information, which tracks and updates the robot’s position in real-time. Under “move_base”, the “global_planner” is responsible for calculating the optimal path from the current location to the destination. The “global_costmap” contains a global cost map of environmental obstacles and navigable areas. The “local_planner” is a local path planner that performs real-time obstacle avoidance and calculates local paths. The “local_costmap” reflects the local cost map of obstacles and navigable areas around the robot. “Recovery_behaviors” handle anomalies and replan paths as needed. The “map_server” provides preconstructed maps for use. “Sensor sources” include sensors, like Lidar and IMU, used for environmental scanning and obstacle detection. Sensor data is published through topics. The “base controller” represents the robot’s base, which moves by subscribing to the velocity commands published to the “/cmd_vel” topic by “move_base”. “tf/tfMessage” processes the transformations between various coordinate frames, ensuring that sensor data and odometry data are processed and applied within a unified coordinate system. For instance, it transforms the Lidar scan data from the Lidar coordinate frame (laser) to the robot’s base coordinate frame (base_footprint), and the IMU coordinate frame (imu_link) to the robot’s base coordinate frame (base_footprint). The “nav_msgs/Odometry” message provides odometry data, including the robot’s position, velocity, and directional information. The “nav_msgs/GetMap” message is used to retrieve map data from the “map_server”. The “sensor_msgs/LaserScan” message retrieves Lidar scan data, including distance information about the surrounding environment. The “geometry_msgs/PoseStamped” and “geometry_msgs/Twist” messages are used to send the target position and orientation (via the “move_base_simple/goal” topic) and the linear and angular velocity commands (via the “cmd_vel” topic) to the base for execution, respectively.

## 3. Experimentation and Results

### 3.1. Experimental Setup

This study involves inference and training of the YOLOv5 model on a PC, based on the PyTorch framework. The specific experimental environment is detailed in Section 2.1. To ensure that the experimental results more accurately reflect the characteristics and true accuracy of the apple target task data, pretrained models are not used. The epoch is set to 200 rounds, the batch size is set to 8, and the image input resolution is 640 × 640 pixels. A stochastic gradient descent optimizer is used, with the other parameters set to their default values as specified in the official YOLOv5 YAML file.

### 3.2. Evaluation Metrics

This study employs the following evaluation metrics: precision (*P*), recall (*R*), mean average precision (*mAP*), model parameter count (parameters), and model computational load (GFLOPS). Precision (*P*) represents the proportion of true positive samples to the total predicted positive samples, while recall (*R*) represents the proportion of true positive samples to the total actual positive samples. The mean average precision (*mAP*) is the average area under the *PR* curve formed by precision and recall. Additionally, the total number of parameters in the model (parameters) and the model’s computational load, measured in giga floating point operations per second (GFLOPS), are included. The formulas for precision, recall, and average precision are as follows:

(15) $$P=\frac{TP}{TP+FP}$$

(16) $$R=\frac{TP}{TP+FN}$$

(17) $$mAP=\frac{\sum_{1}^{N}\int_{0}^{1}P(R)d(R)}{n}$$

where *TP* (true positive) is the number of samples predicted as positive that are actually positive; *FP* (false positive) is the number of samples predicted as positive that are actually negative; *FN* (false negative) is the number of samples predicted as negative that are actually positive.

### 3.3. Comparison of Different YOLOv5 Detection Algorithms

**Figure 10:**
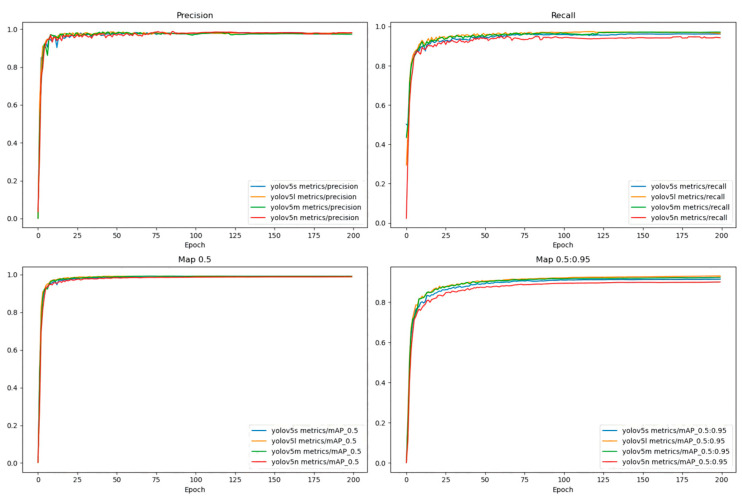
Comparison of different YOLOv5 model metrics.

As shown in Figure 10, 200 training rounds were conducted on the same training set to test the intuitive changes in various metrics for 4 different YOLOv5 models. The precision, recall, and mAP0.5 of the four YOLOv5 models on the apple training dataset have converged, and the differences in mAP0.5 are relatively small.

As shown in Table 3, the YOLOv5 model performs well in apple detection and meets the experimental requirements. This paper selects the YOLOv5n model. It has the advantage of a significantly lower parameter count and computational complexity compared to the other three YOLOv5 models, while also maintaining a high mAP.

**Figure 11:**
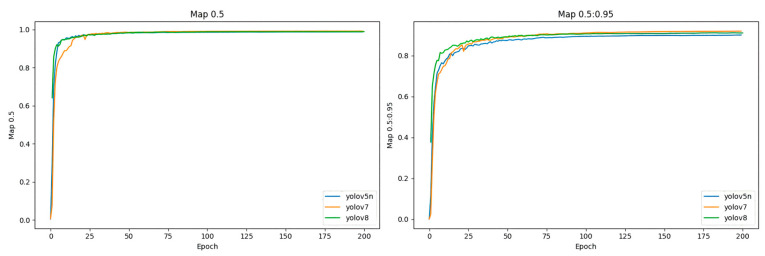
Comparison of mAP metrics for different models.

As shown in Figure 11, to verify the performance of the selected model, a comparison of the performance of 4 different network models over 200 epochs was conducted. The same apple dataset used for the aforementioned YOLOv5 comparison models was utilized for training. Among the four models, the mAP@0.5 values were relatively similar, with YOLOv7 achieving the best performance with the highest accuracy of 99.76%. At the same time, it achieved the highest average precision across different thresholds (mAP@0.5:0.95) with an accuracy of 91.96%. Although YOLOv7 achieved the highest accuracy, its parameter count is as high as 37,196,556 M, which is far greater than the parameter counts of YOLOv5n and YOLOv8. A larger parameter count means it requires more storage space, making it less suitable for resource-constrained mobile devices. On the other hand, its GFLOPS (floating point operations per second) is 105.1, indicating a very high computational complexity. Higher computational complexity results in longer computation times and higher power consumption, which is a significant disadvantage for mobile devices that require high real-time performance.

Based on the data in Table 4, it can be further concluded that YOLOv5n optimizes the number of parameters and computational complexity while maintaining high accuracy. Among the 4 models compared, YOLOv5n has the smallest number of parameters, only 1,765,270 M, which means that it occupies less storage space in resource-constrained situations. Additionally, it has the lowest computational complexity, resulting in faster computation speed, making it suitable for real-time recognition and localization in harvesting robots.

### 3.4. Target Detection Results

**Figure 12:**
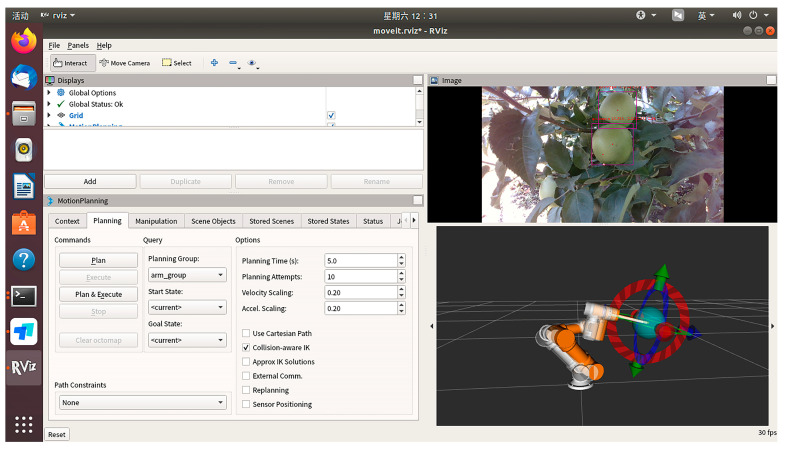
Apple recognition results in the RViz visualization interface.

After assembling the SDK and drivers for the D435i camera in the ROS system, the system subscribes to the color images of apples, aligned depth maps, and camera intrinsic parameters from the camera. Using the formula described in Section 2.4, the spatial coordinates of the apple targets identified by YOLO are calculated. Subsequently, through tf coordinate transformations and communication with the robotic arm, the spatial coordinates of the apples in the base coordinate system of the robotic arm are obtained. Upon activating YOLO and the depth camera, the results are visually displayed in the RViz interface. Figure 12 shows the visualization software interface we use for controlling the robot. This software is based on the ROS framework and integrates the MoveIt! motion planning library to achieve motion planning and control of the robot. The top section of the software includes global options where the global status and coordinate system can be viewed and set. The following steps outline how to configure and display your own robot in the Rviz visualization interface, including creating the robot’s URDF file, setting the robot description parameters, and configuring Rviz to display the robot model. MoveIt! motion planning functionalities are used to implement path planning, path constraints, velocity scaling, and acceleration scaling parameter settings. The top right window of the visualization interface displays real-time images from the camera, processed by YOLOv5 for object detection. This is achieved by subscribing to the camera image topic, processing the image data with YOLOv5, publishing the results, and displaying them in Rviz. The bottom right window provides a three-dimensional view that shows the robot’s posture and motion trajectory in real-time. The advantages of the visualization interface include intuitively understanding the robot’s status and position and observing the YOLOv5 algorithm’s detection results. Additionally, it allows for viewing the robot’s sensor data and motion status for in-depth analysis, improving the debugging efficiency of the robot system. This provides strong support for the application of apple-harvesting robots in complex environments.

### 3.5. System Integration and Control of the Robot

In this study, we used the RPLidar A2 for environmental perception and obstacle avoidance. It provides 360-degree environmental scan data by publishing on the “/scan” topic. The Lidar data are used for constructing environmental maps and real-time obstacle detection. The laser scan data published on the scan topic is crucial for these tasks. The working voltage of the Lidar is 5 V, connected to the power module of the robot chassis. The RealSense D435i camera was employed for object recognition and distance measurement. It acquires color images through the “camera/rgb/image_raw” topic and depth images through the “camera/depth/image_raw” topic. These image data are used to detect apples and calculate their 3D positions and diameters. The camera is connected to the AGX Orin development board via USB 3.0., with a supply voltage of 5 V. The IMU is used for attitude estimation and odometry calibration. It publishes acceleration and angular velocity data through the “imu/data” topic, which helps correct accumulated errors during the robot’s movement. The IMU module is integrated into the chassis controller, with a supply voltage of 5 V. All these sensors are integrated and mounted onto a mobile chassis for autonomous movement. The movement of the chassis is controlled by a chassis driver, which communicates with ROS nodes to achieve power control and path tracking. The chassis is equipped with a 24 V rechargeable battery that powers the entire system. Mounted on the chassis is a six-degree-of-freedom robotic arm with a flexible gripper for harvesting and placing tasks. The flexibility and multiple degrees of freedom allow efficient operation and grabbing of apples. The robotic arm is powered by a 24 V power module and controlled by the AGX Orin development board through ROS. After assembling the hardware, we installed ROS on the Ubuntu 18.04 system and set up the necessary drivers and ROS packages for each sensor. We created a Python virtual environment on Ubuntu that matches the environment used to train the YOLOv5 model on the PC. We loaded the trained .pt file and the YOLOv5 model into the robot operating system (ROS). Additionally, we loaded the navigation algorithms into the ROS to control the apple-harvesting robot and complete its tasks.

### 3.6. Autonomous Navigation and Grasping Results

**Figure 13:**
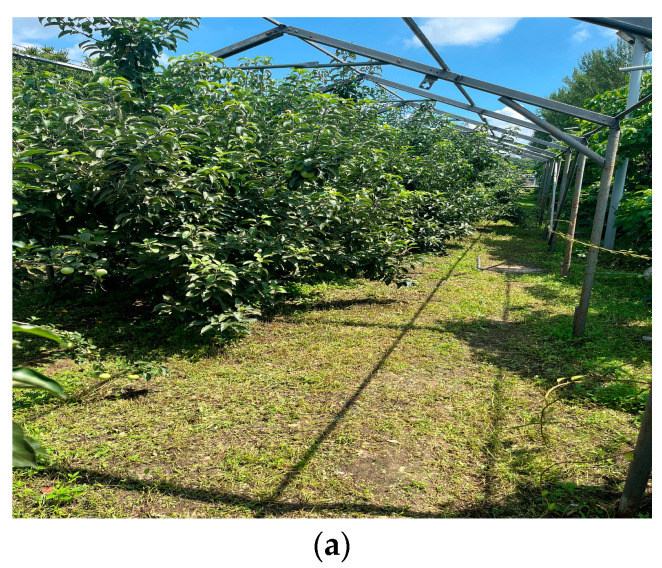

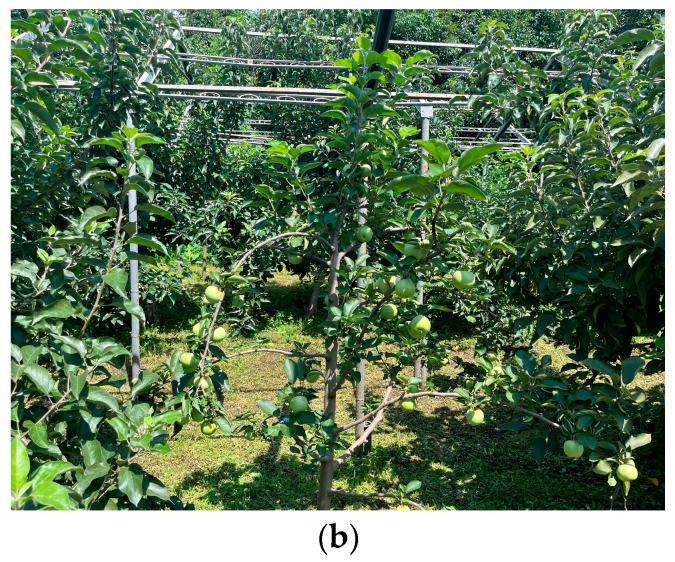
Apple orchard environment. (**a**) An open pathway beside the apple trees in the orchard; (**b**) A frontal view of more apple trees with some apples visible on the branches.

To validate the feasibility and practicality of the apple-harvesting robot’s algorithm, we conducted field tests in an actual apple orchard. Figure 13 showcases the environment of the apple orchard, which served as the test site for the apple-harvesting robot in real world conditions. The field tests aimed to assess the robot’s capabilities in autonomous navigation, obstacle detection, and apple harvesting in a real-world setting.

**Figure 14:**
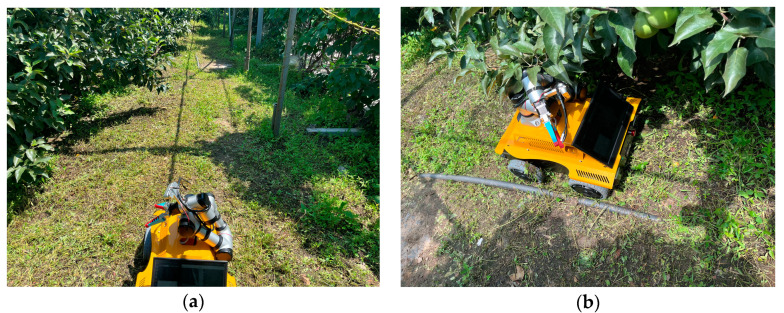

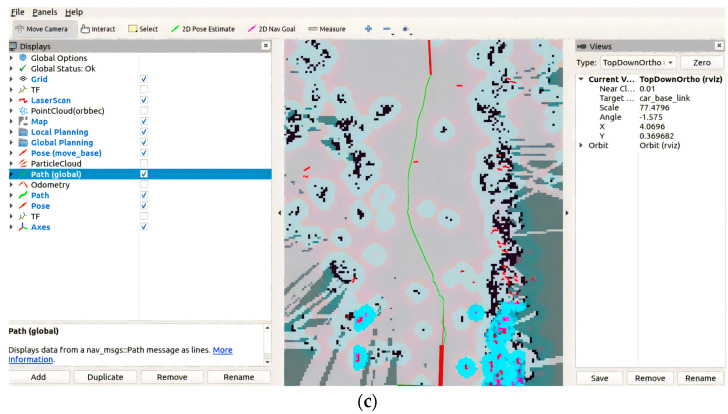
Robot mapping and navigation.

The robot uses Lidar to map the apple orchard environment for autonomous navigation and obstacle detection. Initially, the robot is placed at the starting position in the orchard, and both the Lidar and ROS system are activated. The Lidar begins rotating and emitting laser beams to measure the distances to surrounding objects. The returned laser data, including distance values from various angles, are used to draw a 2D map of the orchard environment. The gmapping algorithm is utilized for mapping, during which the robot continuously moves and updates its position on the map in real-time. The Lidar keeps scanning and integrates new data into the existing map, making it more accurate. The robot employs the adaptive Monte Carlo localization (AMCL) algorithm to determine its position within the map, ensuring precise real-time localization. Once mapping is complete, the robot can use path planning algorithms, such as Dijkstra and TEB, to plan the optimal path from its current location to the target point, dynamically avoiding obstacles during navigation, as shown in Figure 14.

**Figure 15:**
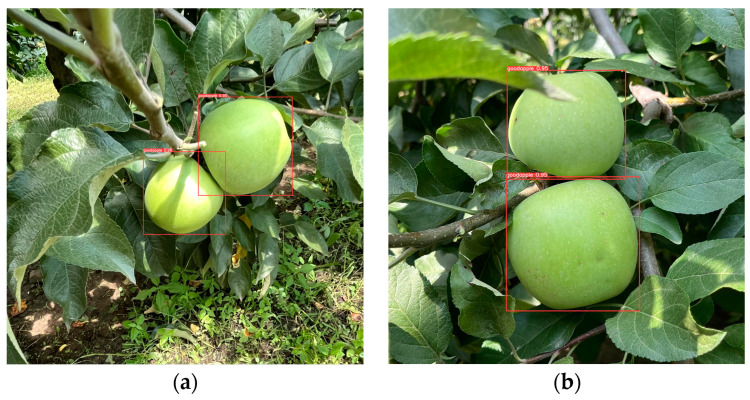
Detection results of orchard apples. (**a**) Detection of two apples distributed on the left and right sides of the tree by the robot, the red bounding boxes indicate the identified apples, labeled as "goodapple" with confidence scores of 0.95 and 0.96; (**b**) Detection of two apples distributed vertically on the tree by the robot, the red bounding boxes indicate the identified apples, labeled as “goodapple” with confidence scores of 0.95 and 0.95.

**Figure 16:**
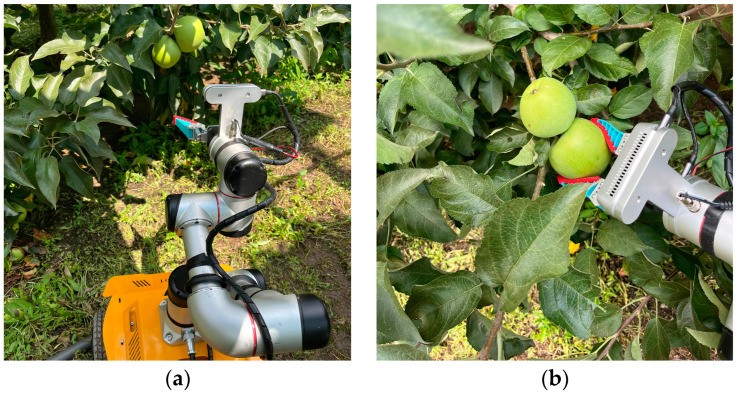
Illustration of the apple harvesting robot in operation. (**a**) The robot’s arm approaching the apples on the tree, getting ready to pick the apples; (**b**) The robot’s gripper carefully grasping the apples, preparing to perform the harvesting task.

After the robot autonomously navigates to the vicinity of the target point, the practical applicability of the detection algorithm was validated through the image recognition and localization of apples. The actual performance test results of the model are shown in Figure 15. The model can detect and recognize apples in the orchard and output coordinate information. The recognition and detection information can drive the apple-harvesting robot to perform the harvesting task, and the robot takes an average of 9 s to pick an apple and return to the initial pose. The performance of the robot in harvesting apples is shown in Figure 16.

**Figure 17:**
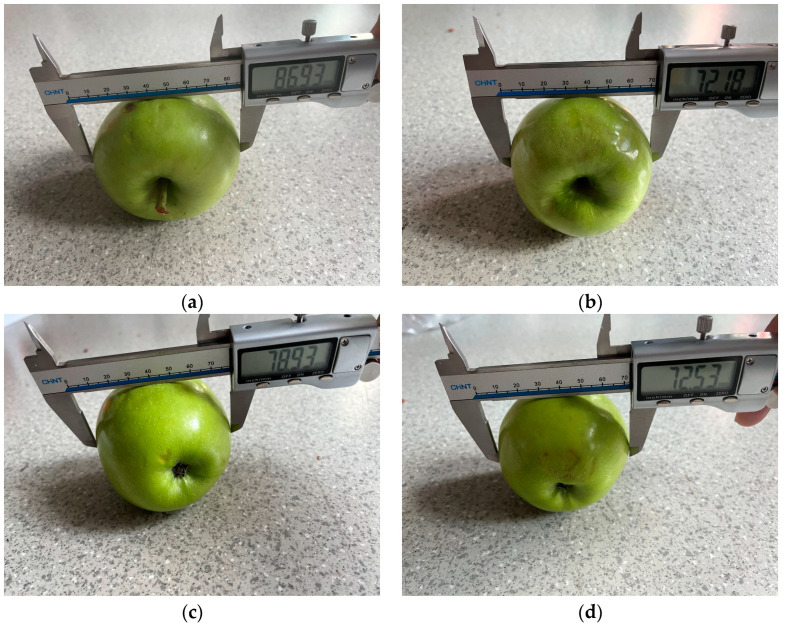
Actual measurement of apple diameter. (**a**) Diameter measurement of apple 1; (**b**) Diameter measurement of apple 2; (**c**) Diameter measurement of apple 3; (**d**) Diameter measurement of apple 4.

To verify the accuracy of the proposed algorithm for calculating apple diameters, the diameter detection data of apples over 50 frames from 2 groups of experiments in the orchard were compared. First, the apples were measured using a standard method, as shown in Figure 17. The stability and error estimation of the diameter measurements were assessed using standard deviation and variance.

**Figure 18:**
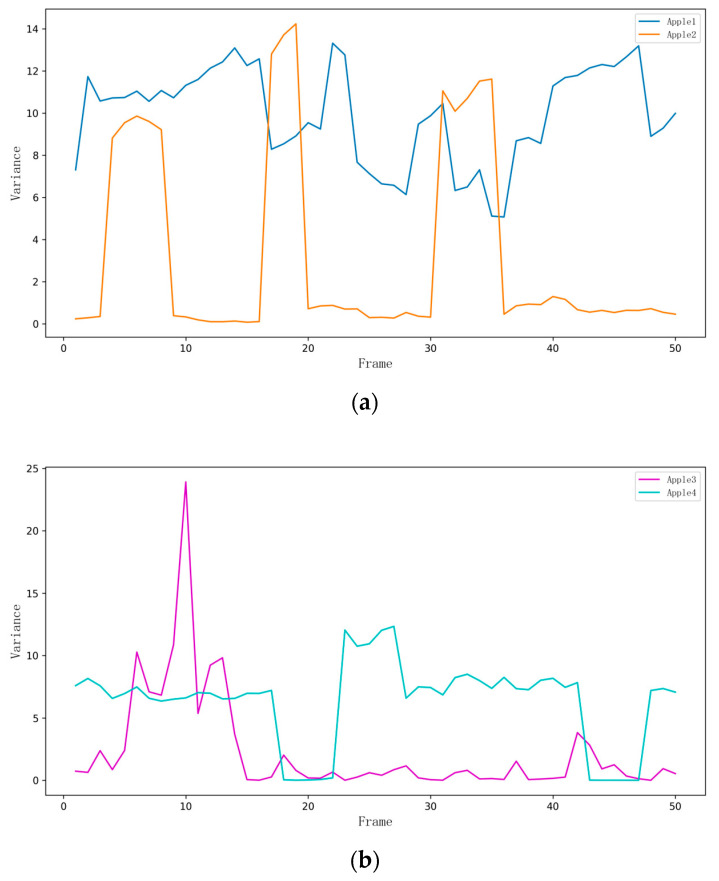
Apple variance change chart. (**a**) The variance in diameter measurements over 50 frames for Apple 1 and Apple 2 shows how the accuracy of the camera in measuring apple diameters fluctuates over time; (**b**) The variance in diameter measurements over 50 frames for Apple 3 and Apple 4 shows how the accuracy of the camera in measuring apple diameters fluctuates over time.

Through the analysis of variance, it can be seen that the accuracy of the camera in measuring the diameter of apples fluctuates between different frames, as shown in Figure 18.

Table 5 shows the diameter data of different orchard apple samples measured using both actual and camera methods and evaluates the accuracy and stability of these measurements using standard deviation and variance. The standard deviation represents the difference between the actual measurement value and the camera measurement value, while the variance indicates the degree of dispersion of the camera measurement values relative to the actual measurement values. Overall, the camera measurement values are generally lower than the actual measurement values, with errors ranging from 1.12 mm to 3.13 mm.

## 4. Discussion

In the orchard environment, we conducted practical experiments on mapping, navigation, apple picking, and the measurement of actual diameters. The experiments included the following steps. We used the RPLidar A2 laser scanner to map the orchard environment. To conserve the robot’s battery, the robotic arm was not activated during the mapping process. We utilized the gmapping algorithm to construct a 2D map of the orchard. This algorithm uses particle filter technology, which provides high mapping accuracy while maintaining low computational requirements. Experimental results show that the map meets the requirements for autonomous navigation of the robot. After completing the mapping, the robot used the Dijkstra algorithm for global path planning, combined with the TEB (timed elastic band) local path planning algorithm for dynamic obstacle avoidance. Experimental results indicate that the robot can autonomously navigate in the orchard environment, accurately avoid obstacles, and successfully reach the target location. This validates the effectiveness and robustness of our navigation algorithm. Upon reaching the target location, the robot used the YOLOv5-RACF algorithm for apple recognition, localization, and diameter measurement. The average processing time for the YOLOv5-RACF algorithm to detect and estimate apple diameter was approximately 0.14 s. In different experimental groups, by comparing actual measurements with camera measurements, it was found that the measurement error ranged from 1 to 3 mm. This demonstrates that the algorithm is highly efficient in terms of real-time performance, meeting the real-time detection requirements in practical applications. The robot then used its mechanical arm to pick apples. Experimental results show that the average time for the robot to pick an apple and return to the initial position was 9 s. The study identified two limitations during the apple picking process: (1) Despite various techniques to reduce the robot’s positioning error and the use of a PID controller to minimize the error between the preset position and the current position, the complex terrain of the orchard still caused wheel slippage, resulting in a deviation between the actual position and the expected position. (2) The presence of mutual occlusion between apples and occlusion by branches and leaves posed challenges for recognition, picking, and diameter measurement of the apples.

To improve the performance and applicability of the apple-picking robot, future research should focus on the following areas: (1) Developing more advanced control algorithms to enhance the robot’s stability and positioning accuracy on uneven terrain. (2) Implementing more complex image processing techniques and sensor fusion strategies to improve apple detection and measurement accuracy in the presence of occlusions. (3) Exploring energy-saving strategies for the robot’s components, particularly during extensive field operations, to extend battery life and operational duration. (4) Integrating additional sensors, such as imaging cameras or multispectral sensors, to enhance the robot’s detection capabilities and accuracy. (5) Conducting more extensive field tests in different orchard environments and seasons to validate the robot’s performance and adaptability under various conditions.

## 5. Conclusions

In this work, we studied an apple-harvesting robot that integrates deep learning and image processing technologies with the ROS system. The robot achieves autonomous navigation in orchard environments, real-time detection and recognition, and precise diameter measurement for apple harvesting. This robotic technology represents a significant advancement in agricultural automation, demonstrating the feasibility of automated apple harvesting, which can effectively alleviate labor shortages and improve production efficiency. However, there are still some deficiencies and areas for further research in the experiment. For instance, navigation accuracy needs improvement in complex orchard environments. The depth camera’s apple recognition is affected by fruit occlusion, branch and leaf obstruction, and unstable light intensity, which are challenges for the automatic harvesting of apples by agricultural robots. Therefore, future work can focus on improving navigation algorithms and enhancing the recognition of apple targets under occluded conditions to increase the robustness of the apple-harvesting robot, making it adaptable to more complex agricultural environments.

## Figures and Tables

**Table 1:** Detailed information of the apple dataset.

Label	Original Image Data	Augmented Image Data	Training	Validation
goodapple	3070	6864	8952	982

**Table 2:** List of parameters for the path navigation algorithm.

teb_local_planner_params	global_costmap_params	local_costmap_params
dt_ref: 0.3 max_vel_x: 0.5 min_vel_theta: 0.2 acc_lim_theta: 3.2 yaw_goal_tolerance: 0.1 inflation_dist: 0.6 obstacle_poses_affected: 30 weight_kinematics_forward_drive: 1000 weight_optimaltime: 1 weight_dynamic_obstacle: 10 weight_preferred_direction: 0.5	global_frame:/map update_frequency: 1.0 transform_tolerance: 0.5 inflation_radius: 0.6	global_frame:/odom update_frequency: 5.0 transform_tolerance: 0.5 inflation_radius: 0.6
dt_hysteresis: 0.1 max_vel_theta: 1.0 acc_lim_x: 2.5 xy_goal_tolerance: 0.2 min_obstacle_dist: 0.5 costmap_obstacles_behind_robot_dist: 1.0 weight_kinematics_nh: 1000 weight_kinematics_turning_radius: 1 weight_obstacle: 50 weight_viapoint: 1 weight_adapt_factor: 2	robot_base_frame:/base_footprint publish_frequency: 0.5 cost_scaling_factor: 5.0	robot_base_frame:/base_footprint publish_frequency: 2.0 cost_scaling_factor: 5.0

**Table 3:** Comparison of the results of different YOLOv5 network models.

Network	P/%	R/%	mAP@0.5/%	mAP@0.5:0.95/%	Parameters/M	GFLOPS
yolov5s	97.978	96.144	99.051	91.409	7,022,326	15.9
yolov5l	98.042	96.56	99.144	92.99	46,138,294	108.2
yolov5m	97.335	97.129	99.133	92.312	20,871,318	48.2
yolov5n	98.131	94.272	98.748	90.02	1,765,270	4.2

**Table 4:** Comparison of the results of different network models.

Network	P/%	R/%	mAP@0.5/%	mAP@0.5:0.95/%	Parameters/M	GFLOPS
Yolov5n	98.131	94.272	98.748	90.02	1,765,270	4.2
Yolov7	96.77	97.53	99.22	91.96	37,196,556	105.1
Yolov8	98.204	95.08	98.902	91.017	3,011,043	8.2

**Table 5:** Analysis of apple diameter measurement results.

Object	Actual Measurement Value/mm	Camera Measurement Value/mm	Standard Deviation/mm	Variance/mm^2^
Apple1	86.93	83.792108	3.127892	9.924321
Apple2	72.18	70.813700	1.366300	3.237878
Apple3	78.93	77.812189	1.117811	2.324057
Apple4	72.53	70.320186	2.255963	6.267101

## Data Availability

The datasets generated and/or analyzed during the current study are available from the corresponding author upon reasonable request.
